# A guide for the use of fNIRS in microcephaly associated to congenital Zika virus infection

**DOI:** 10.1038/s41598-021-97450-w

**Published:** 2021-09-29

**Authors:** João Ricardo Sato, Claudinei Eduardo Biazoli Junior, Elidianne Layanne Medeiros de Araújo, Júlia de Souza Rodrigues, Suellen Marinho Andrade

**Affiliations:** 1 Center of Mathematics, Computing, and Cognition, Federal University of ABC, São Bernardo do Campo, SP Brazil; 2 Department of Biological and Experimental Psychology, Queen Mary University of London, London, UK; 3 Laboratory of Aging and Neuroscience Studies, Department of Physical Therapy, Health Sciences Center, Federal University of Paraíba, João Pessoa, PA Brazil; 4 Department of Preventive Medicine, University of São Paulo, São Paulo, SP Brazil

**Keywords:** Cognitive neuroscience, Diseases of the nervous system

## Abstract

Congenital Zika Syndrome (CZS) is characterized by changes in cranial morphology associated with heterogeneous neurological manifestations and cognitive and behavioral impairments. In this syndrome, longitudinal neuroimaging could help clinicians to predict developmental trajectories of children and tailor treatment plans accordingly. However, regularly acquiring magnetic resonance imaging (MRI) has several shortcomings besides cost, particularly those associated with childrens' clinical presentation as sensitivity to environmental stimuli. The indirect monitoring of local neural activity by non-invasive functional near-infrared spectroscopy (fNIRS) technique can be a useful alternative for longitudinally accessing the brain function in children with CZS. In order to provide a common framework for advancing longitudinal neuroimaging assessment, we propose a principled guideline for fNIRS acquisition and analyses in children with neurodevelopmental disorders. Based on our experience on collecting fNIRS data in children with CZS we emphasize the methodological challenges, such as clinical characteristics of the sample, desensitization, movement artifacts and environment control, as well as suggestions for tackling such challenges. Finally, metrics based on fNIRS can be associated with established clinical metrics, thereby opening possibilities for exploring this tool as a long-term predictor when assessing the effectiveness of treatments aimed at children with severe neurodevelopmental disorders.

## Introduction

Zika virus is a flavivirus, in the family Flaviviridae transmitted by mosquitoes of the genus Aedes sp. Initially, there was no evidence for the virus causing human disease. However, an outbreak on several islands in the State of Yap, Federated States of Micronesia in 2007 resulted in an estimated 5000 infections among the total population of 6700 individuals^1, 2^. Subsequent outbreaks, in 2013 and 2014, in French Polynesia, show cases with asthenia, wrist and fingers arthralgia, conjunctivitis, headache, rash associated with positive results of RT-PCR for ZIKV^3, 4^. In March 2014, Oehler et al. described the first case of Guillain-Barré syndrome attributable to ZIKV and reported that, since the beginning of the Zika virus epidemic, the incidence of Guillain–Barré syndrome in French Polynesia has increased about 20 times^5^. In October 2015, the first cases of fetal microcephaly associated with ZIKV infection during pregnancy were observed in Pernambuco State, Brazil^6^. In December 2015, the World Health Organization (WHO) issued a warning for this congenital anomaly, as well as for acute neurological syndrome, both plausible associated with ZIKV during pregnancy^7^.

Congenital Zika Syndrome (CZS) is characterized by changes in cranial morphology associated with heterogeneous neurological manifestations and cognitive and behavioral impairments. CZS clinical impairments can last and dynamically change over development^8^. Structural neuroimage findings in newborns with CZS include ventriculomegaly (mainly in the posterior portions of the lateral ventricles), abnormalities of the corpus callosum (hypoplasia or hypogenesis), demyelination and hypoplasia of the cerebellum and brainstem^9^. Moreover, there is compelling evidence for increased incidence of focal and multifocal epileptiform discharge patterns in these patients^10^. Irritability and hypersensitivity to touch are usual features of infants with CZS and microcephaly, greatly hampering any physical examination procedure. Furthermore any stimulation can lead to exacerbation of motor signs and symptoms such as hyperexcitability (clonus following stimulation), or trigger partial or generalized seizures. In this way, continuous monitoring of children with desensitization strategies during any clinical procedure is of paramount importance not only for the child's well-being but also to ensure the evaluation's quality^11–15^.

Structural and functional neuroimaging studies might be critical tools to inform long-lasting clinical care of CZS patients. Besides identifying baseline abnormalities, neuroimaging investigations during longitudinal follow-up can be potentially used to better predict the prognosis and evolution of children with CSZ. Although repeated follow-up Magnetic Resonance Imaging (MRI) acquisitions could potentially provide clinically significant information to guide care and recovery strategies, important shortcomings preclude the use of frequent MRI in children with CZS. The environmental characteristics of MRI scanning such as loud noises, maintenance of supine positions and restrictions to head movement justify the necessity of sedating sensitive pediatric patients^16^. However, the risk of worsening neurological impairment by repeatedly sedating children for follow-up neuroimaging studies would far outweigh the potential benefits. Besides, available protocols to avoid sedation in pediatric patients undergoing MRI scanning^17^ would not be feasibly applied for most children with CZS given the clinical features highlighted above. Added to the relatively high cost of MRI, these characteristics foreclose the possibility of applying this neuroimaging modality for the longitudinal follow up of children with CZS and microcephaly.

In contrast, fNIRS is a low-cost technique, less susceptible to movement artifacts and that can be acquired in environments suitable and adapted to sensitive pediatric patients without requiring sedation. Hence, fNRIS is a feasible neuroimaging modality for longitudinal studies of children with CZS. fNIRS measures are based on the fact that neural activity is accompanied by changes in blood oxygenation, brain volume and blood flow. Thus, the absorption of near-infrared light by these blood chromophores (i.e.,oxy and deoxyhemoglobin) enables one to record the relative changes in their concentrations and indirectly monitor local neural activity^18^. Several studies have used fNIRS as a tool to investigate brain functional alterations in children with neurological and cognitive dysfunctions in recent years^19–21^. However, the feasibility and potentiality of measuring neuro-hemodynamic activity during development in patients with microcephaly associated with the Zika virus remains to be investigated. To the best of our knowledge, no studies to date have recorded fNIRS signals on children with CZV.

In the current report, we share our experience in acquiring fNIRS data of children diagnosed with microcephaly related to CZS. From this experience, we propose a guideline which aims to provide a potential framework for building methodological guides for fNIRS acquisition and analyses in children with neurodevelopmental disorders. When preparing for this study, our team faced and recognized several methodological challenges including: (1) difficulty in handling the cap due to patient hypersensitivity and (2) adapting its size to the reduced head circumference; (3) high prevalence of low visual acuity; (4) hypotonia and episodes of spasms with possible seizures; and (5) the presence of certain phenotypic characteristics such as pigmentation, volume and capillary structure that can directly impact fNIRS signal-to-noise ratio^22, 23^. Here we systematically describe the methodological shortcomings specific to this population and potential ways to overcome them. Such methodological issues and guidance were accessed for the stages of: (1) pre-collection (preparation and desensitization of participants, choosing fNIRS setup; (2) fNIRS scanning and (3) post-collection (data pre-processing and analysis strategies). Finally, we discuss the perspectives, challenges and limitations in fNIRS data acquisition in pediatric neurological clinical populations during development.

## Results

### Suggested protocol

Children with microcephaly and congenital ZKV must undergo gradual preparation and desensitization procedures in order to optimize the conduct of procedures with fNIRS due to hypersensitivity to both touch and any foreign object around the head. These participants present specific behavioral impairments such as reacting to sound and visual stimuli differently than healthy controls^24^, and which implies the use of playful resources to favor their adaptation. From the present experience, we suggest conducting a systematized protocol involving the following considerations.

#### Materials and tolerance to stimuli

As the first step of the experiment, we suggest defining which materials can be used as reinforcements for the child such as protocols which have already been endorsed for other pediatric populations^25^. We chose materials of a playful nature in our study and interviewed the parents about which types of toys would have a positive value for children in order to be used during the experiments. Three toys were selected and presented to the child over a period of 10 min for free handling. It was observed which of the toys the child handled/played with the most, which was then categorized as the one with the greatest valence.

Categorizing valences can be a useful strategy when the child enters the room where the experiment will be conducted, as well as to extend the acquisition time, reducing the participant’s fatigue and irritability levels. Reinforces can then be offered to the child during each desensitization session and subsequent fNIRS experimental sessions, and the valence scale follows the order of presentation, meaning that as the greatest discomfort is mentioned, the reinforcer will be changed, alternating the valences and favoring acceptance of the procedures by the participant.

The people responsible for these adaptations in our study were members of the experimentation team who positioned themselves next to the child during fNIRS acquisition. We observed that familiar faces, both of the parents and of the experimenters, can represent a facilitator during the session. Importantly, elements which can be configured as negative should be taken into consideration and excluded from the environment before the acquisition, whenever possible.

#### fNIRS cap

The desensitization process must include the child’s adaptation to the use of the fNIRS cap, as well as becoming familiarized with the type of task or stimulus that will eventually be applied. The use of a cap with flexible and comfortable material and also presented in a playful manner is of paramount importance. We used a cap made of elastic neoprene material which was well-adjusted to the head and without optodes to start the familiarization process with the fNIRS equipment in our experiment, right after the familiarization with the reinforcers.

It is necessary to consider that because touch in the vicinity of the cephalic region is considered an aversive stimulus for many of these children, tissue adaptation can be a crucial factor for acceptance of the fNIRS cap. Hypersensitivity to touch and hyperreflexia are common clinical findings in this population, and are often accompanied by excessive crying^26^, which makes it even more difficult to perform tests demanding body manipulation.

Preliminary evidence suggests that the response time is inversely proportional to the amount of information presented in a single sensory channel and that the amount of information acquired by a neuron gradually increases with time. In parallel, it is assumed that a multisensory stimulus can anticipate responses when compared to a unisensory stimulus^27^. In our experience, we used principles of sensory integration therapy before putting on the cap by following the steps of sensory recording; guidance and attention; interpretation (meaning of the stimulus for the child); organization of the response and execution of the response^28^. Thus, the child begins to adapt to pressure and touch stimuli in this region through multiple and stable tactile stimuli, which progressively cover the entire head circumference. We suggest placing the optodes after using the cap for 10 min as well as using the reinforcers during this process, which are the toys of greater value so that the child does not recognize this new stimulus as aversive.

We note that it is important to take into account that control at each stage must be achieved before proceeding to the next stage. The child might experience discomfort at each new level and therefore optodes placement must be gradual; furthermore, if two caps are used, one for adaptation and one with the optodes, the experimenter must plan the adaptation time and the desensitization procedures inherent to each procedure.

#### Optodes placement

An individual montage for fNIRS optodes placement is an important step in CZS children, since the size and shape of the head and brain are abnormal. Therefore, it is highly recommended, besides the 10–20 system, to use additional individualized localization techniques using the structural MRI information and a 3D digitizer for neuronavigation. When this system is available, it is possible to combine the structural MRI information with anatomical landmarks for a more accurate optodes placement.

#### Signal preprocessing

In addition to the difficulties regarding fNIRS signal acquisition, there are also methodological challenges to take into consideration during preprocessing and analysis. Even though fNIRS is relatively robust against motion artifacts when compared to other methods^29^, usually the signal quality is lower in microcephaly patients when compared to health subjects. Specific features such as involuntary movements and sub-optimal cap adjustment oftently lead to lower signal-to-noise ratio, mainly due to motion induced artifacts. The atypical head thus, motion correction is a mandatory preprocessing step in this population. To the best of our knowledge, the approaches based on regressing out the first principal components from the signals and the wavelet based despiking/discontinuity methods are still the state-of-the-art approaches for motion correction. Based on the study of^30^, we recommend the latter. However, we highly recommend a visual inspection for each subject and in some extreme cases, data exclusion. In these cases, the data quality may be insufficient for motion correction and a more conservative choice is to discard the whole acquisition of a subject. On the other hand, it is important to mention that the impact of motion artifacts is subject dependent and actually, in cases of severe CZS it is also negligible because the subject cannot even move.

In addition, as the presence cardiac artifact in oxyhemoglobin signals is an indicator of good quality, in which this artifact is not present and with high variability must be discarded before further analyses. Frequency band-pass filtering is also a necessary preprocessing step in order to reduce the influence of systemic low (e.g.: trends) and high frequency artifacts (e.g.: cardiac cycle). The cut-off frequencies specification depends on the temporal features of stimuli being presented in the experiment but are usually set between 0.001 and 0.1 Hz. Finally, if short-distance measurements are available, they can be regressed-out from the signals of long-distance channels, aiming to reduce the systemic and superficial artifacts impact in further analyses.

#### Data analysis

Since most young patients are not able to accomplish experiments based on tasks (and even flicking checkerboard might result in seizures), brain mapping based on the General Linear Model on task design is not feasible. Thus, analytical methods focused on resting state fNIRS data are potential alternatives. From these, we illustrate two approaches: functional connectivity (FC) analysis and fractional amplitude of low frequency fluctuations (fALFF) analysis.

Two spatially remote brain regions are functionally connected if they present a temporal correlated activity^31^. In fNIRS studies, this link is investigated by evaluating the temporal dependence among signals of distinct channels. There are many methods to quantify this dependence, but the most used are variations of correlation analyses (in time or frequency domain). When considering a set of brain regions and its pairwise connections, it is also possible to model this system as a graph, a mathematical framework for network analyses^32^. From this perspective, it is possible to identify central regions in a particular network which may be related to cognitive, behavioral or mental health outcomes^33^.

Fractional amplitude of low frequency fluctuations (fALFF) analysis is a method focused on the exploration of spontaneous regional activity^34^. This approach was first proposed to the analysis of functional magnetic resonance imaging (fMRI) data based on the blood oxygenation level dependent signal. Hence functional connectivity, fALFF coefficients and brain regions network centralities can be usually calculated for each subject's resting state fNIRS data. Group statistical analysis comparing groups (e.g.: controls vs. patients) or testing associations between fNIRS features and behavioral scores (e.g.: neurological, psychiatric or neuropsychological assessment) might be conducted. These analyses may be parametric or non-parametric depending on the sample size and violations of Gaussian distribution assumptions.

We illustrate the experimentation and fNIRS data acquisition from a 4 years old female patient with microcephaly (moderated impairment).

**Figure 1:**
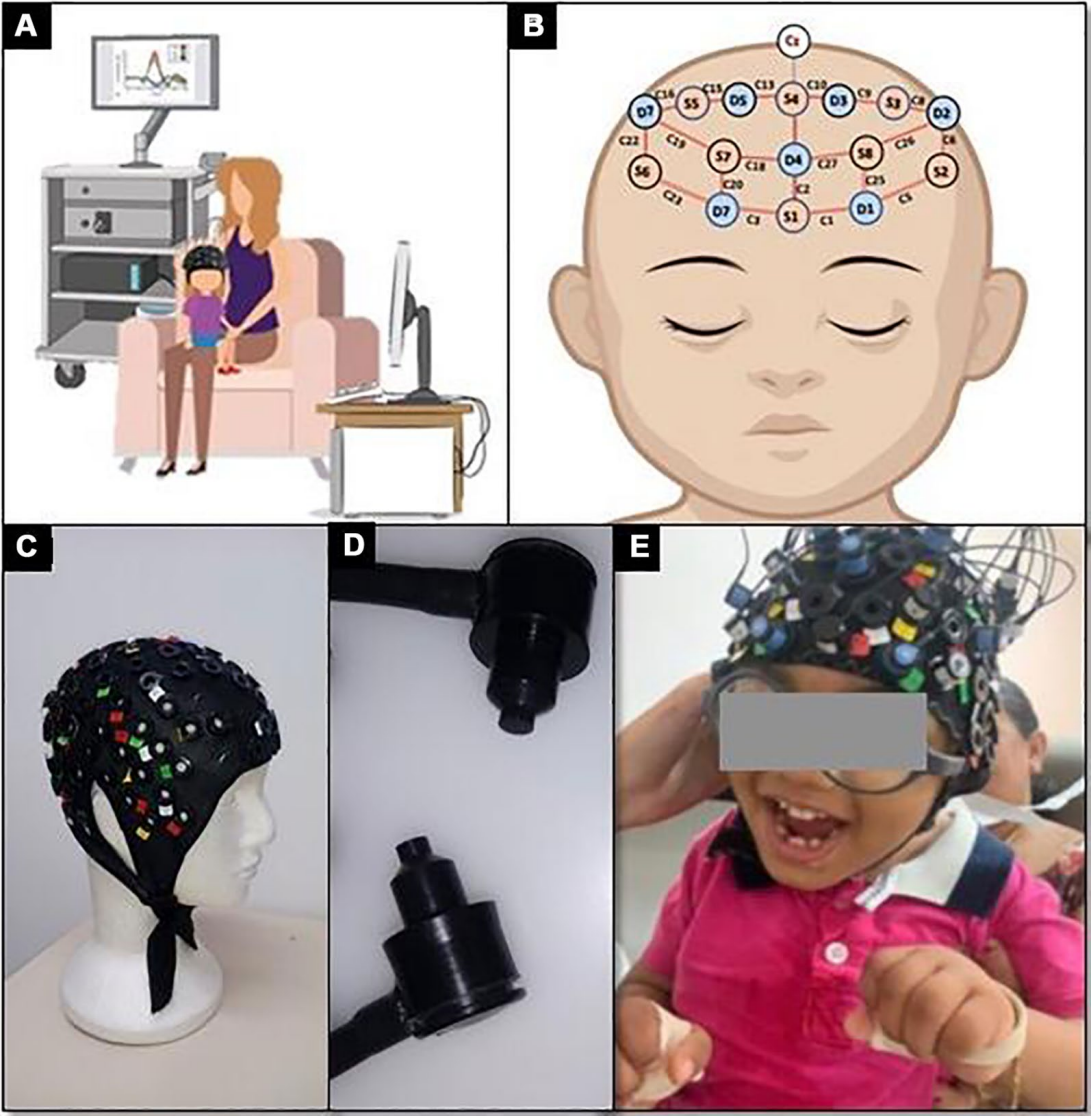
Experimental fNIRS protocol: (**A**) In the experiment, the child used the fNIRS cap and sat on the caregiver's lap while the stimuli were presented by a monitor. The table with fNIRS instrumentation was set at the backside of the armchair, out of the child's visual field. (**B**) The fNIRS topographic layout was composed by 8 pair of optodes (source in red and detector in blue) placed on on the frontal area in the cap. The lines between each source-detector pair in the figure represent the twenty long distance channels. (**C**) fNIRS cap model similar to that used in the experiment. (**D**) fNIRS detectors. (**E**) Illustration of fNIRS in a patient during the execution of the experimental protocol.

The stimuli presented was the Inscapes video described previously (duration of 7:16 min), with simultaneous fNIRS and video recording acquisition. Video recording the experimental session was essential to evaluate the arousal level, amount and amplitude of head movement, evidence for attending to stimuli and to register events of potential clinical relevance (e.g. seizures or spasticity). In this illustrative case, it was possible to observe that the child remained calm and still during most of the acquisition time. During the first 160 s, she attended the movie stimulus presented on a mobile screen, evidenced by gaze maintenance. Using a mobile to present the stimuli was chosen given that our sample are more familiar with mobile than with computer screens and because distance to the stimulus can be easily adapted according to visual impairment. At 160 s of session, an event characterized by a major forward head movement followed by tonic extension of upper and then lower limbs for 13 s was observed. After this event, her gaze wanders for approximately 45 s before returning to the initial position. The same stereotyped event of neck flexion followed by upper and then lower limbs extension was observed at 280 s of the recording, during 9 s in total and being followed by an extended period (~ 50 s) of wandering gaze. Video recording was also important to register physiological movement (e.g. yawning) that can be used to better inform preprocessing^35^ (Fig. 1).

**Figure 2:**
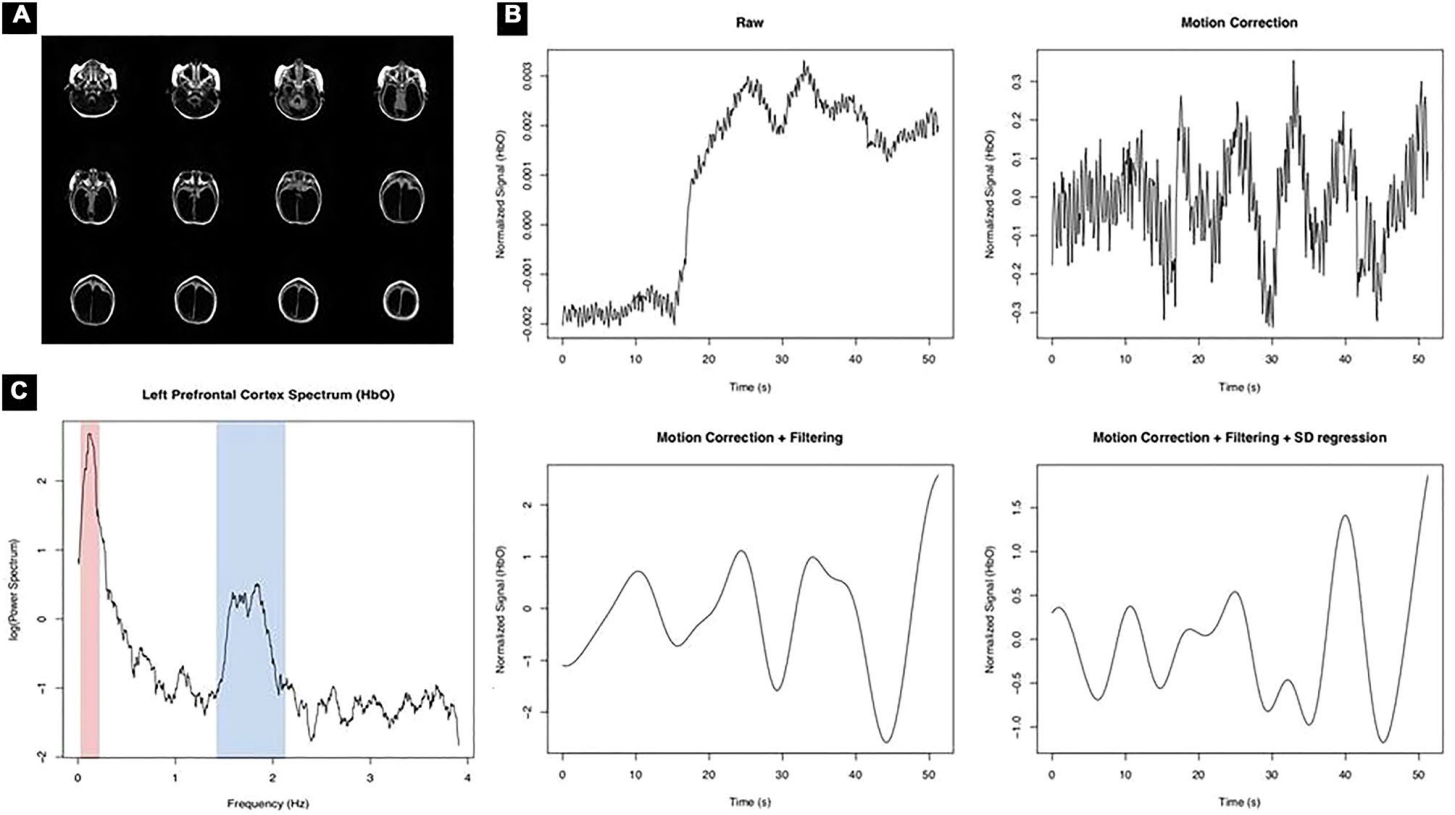
Outcomes from the illustration case: (**A**) Axial Flair T2 MR Image. Radiological Notation. (**B**) HbO signal outcome according each preprocess step: raw data; motion correction; motion correction + filtering; motion correction + filtering + SD regression. (**C**) Power spectrum of a left PFC HbO signal after motion correction. The red and blue shadows highlight the low frequency band (fALFF) and the cardiac artifact band, respectively.

The fNIRS data acquisition was conducted using a NIRSports system (NIRx, USA) with eight sources (two wavelengths, 760 and 850 nm) and eight detectors. The temporal sampling rate was 7.81 Hz. One of the detectors was used with a proper adapter to obtain short-distance channels for each source. The optodes placement was chosen based on the individual MRI observation, taking into account the region with more potential to acquire cortical measures (Fig. 2A). Thus, the optodes were placed according to a prefrontal montage using a 10–10 EEG system cap (Easycap, Germany), resulting in 20 long-distance channels. Unfortunately, an appropriate hardware for integrating MRI anatomical landmarks and 3D digitizer was not available for this acquisition. We acknowledge this is a limitation of this illustrative example.

Figure 2B depicts the preprocessing outcomes from an excerpt of an HbO signal from a left prefrontal channel. The preprocessed data at each step are also shown in this Figure. In the raw data panel, two artifacts are pronounced: a motion artifact around 15 s and also the cardiac artifact over the whole signal (high frequency oscillations). The preprocessed signal by motion correction using wavelets is presented top-right, showing that the discontinuity due to movement was smoothed. At bottom-left, we depicted the signal after band filtering (from 0.01 to 0.1 Hz) and the panel at bottom-right the signal after regressing out the 8 short-distance channels signals (i.e.: the residuals of a multiple regression using the 8 signals as regressors).

In the following, Fig. 2C illustrates the power spectrum of a left PFC HbO signal (after motion correction only) from the patient. The red and blue shadows highlight the low frequency band (fALFF) and the cardiac artifact band, respectively.

**Figure 3:**
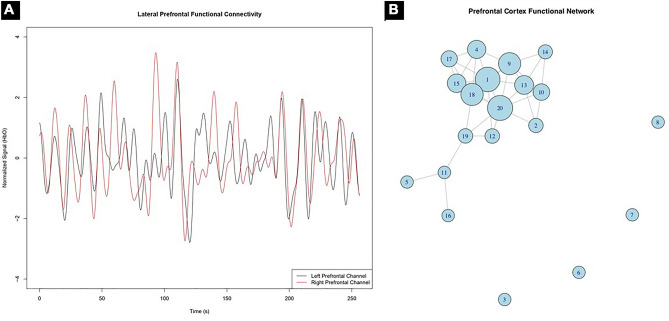
HbO signal outcomes from the illustration case: (**A**) Preprocessed signal from left and right prefrontal channels. Note that the fluctuations along time are similar between hemispheres. The Spearman correlation coefficient correspondent is 0.35 (*p* < 0.001). (**B**) Functional connectivity of 20 PFC channels. Each circle and edge represent a channel and the functional links, respectively. The size of the circle is proportional to the eigenvector centrality of each node (i.e.: the relevance according to the network structure organization).

In Fig. 3A, we show the preprocessed signal from left and right prefrontal HbO channels. Note that the temporal fluctuations are moderately similar, with a Spearman correlation coefficient (functional connectivity) equal to 0.35 (*p* < 0.001; circular-bootstrap method due to signals autocorrelation).

The pairwise HbO signals correlation among the twenty long-distance PFC channels are then calculated, resulting in a 20 × 20 dimension Spearman correlation matrix. This matrix is then binarized at 0.15 and used as the adjacency matrix to model an undirected graph. This functional connectivity graph is illustrated (all PFC channels HbO) in Fig. 3B. Each circle and edge represent a channel (i.e., a brain region) and the functional links, respectively. The radius is proportional to the eigenvector centrality of each node (i.e.: the relevance according to the network structure organization). Note that from this perspective, channels 1 and 20, both located at the left medial orbitofrontal cortex are the most central in this network.

## Discussion

The relatively low cost of acquisition, portability and feasibility of fNIRS resting-state studies in relation to MRI scanning can foster richly-sampled longitudinal studies of children with congenital ZKV syndrome. Longitudinal design is crucial to unveil developmental changes in brain function with relevant behavioral and clinical correlations. The possibility of characterizing local alterations with resting-state paradigms, using fALFF or HRF measures for example, can be potentially used as a proxy for local structural alterations. In this way, longitudinal assessment of localized alterations can be followed-up without the necessity of expensive and stressful repeated MRI scannings.

In this perspective, studies suggest the fNIRS as an alternative and promising tool in the assessment of changes in the neurological course, hemodynamic aspects, identification of structural lesions, as well as in the identification of other CNS changes, according to the impairment of the child's outcomes^20, 36, 37^, mainly because it does not present major movement restrictions, making it suitable for investigations in children in early childhood with behavioral disorders, difficulty in understanding commands and spastic episodes^38, 39^.

The possibility of non-invasively monitoring the hemodynamic response to brain activation during motor and cognitive tasks suggest ways in which fNIRS may be particularly applicable to investigate clinical and functional outcomes in the pediatric population^40, 41^. However, there are still no studies available investigating the effects of the congenital Zika virus, recognized for its teratogenic role, in children with microcephaly using fNIRS. Thus, important questions remain open: how does a brain with multiple injuries and considerable loss of brain tissue functionally interpret external stimuli? In addition, how do oxygen demands or utilization correlate with long-term clinical prognosis?

Understanding these gaps in light of the specific severity of the clinical and cognitive impairment for children with microcephaly, or differentiating responses between patients with mild, moderate and severe disorders can be useful for both planning appropriate rehabilitation programs and for defining clinical metrics which assess synaptic plasticity.

While neuroimaging techniques represent a powerful tool to investigate brain function in a non-invasive and objective way, most of these techniques impose physical constraints on realistic situations. One of the major advantages of fNIRS is the possibility to monitor larger portions of the head on freely moving participants^42^. For example, physical or behavioral therapies cannot be conducted in an MRI scanner, thus fNIRS provides an amazing opportunity to evaluate the efficacy of a specific therapy in CZS kids.

In addition to children with inadequate brain development due to prenatal exposure to Zika Virus, fNIRS has been used to assess the degree of impairment of patients in other neurological disorders that also have microcephaly as one of their clinical manifestations. Despite the limited research involving fNIRS and microcephaly, Bertachini et al. (2021) compared the neuro hemodynamic responses between children with microcephaly associated with Toxoplasmosis and healthy infants, and observed an individual pattern of heterogeneous brain activation in the temporal, parietal and frontal regions in both hemispheres, in response to auditory stimuli^43^.

Similar to what happens with other conditions such as autism and cerebral palsy, the traditionally-used physical therapy involves applying different tasks until the patient can respond positively to executing a module or set of specific demands which help him to reduce his motor and cognitive impairment^40^. If the patients’ hemodynamic responses can be monitored and simultaneously evaluated during task execution, we offer the possibility of systematized guidance both for the conduct of therapy and for its planning. The treatment is no longer focused on the disease and starts to be performed according to the patient’s needs, meaning individually. In this sense, the combined investigation of fNIRS, kinematics and cognitive-behavioral responses to a motor stimulus or a simultaneous interaction with another child can favor developing therapies which are synchronized according to the task, supporting the motor, cognitive and Social processes.

Regarding the scope of social relations in view of the important cognitive-behavioral impairment that most children with congenital ZKV present, understanding individual differences in the therapy planning phase before performing tasks and presenting stimuli becomes crucial to successful treatment. In addition, we know that the damage caused by the Zika virus can remain for a long time, and that associated comorbidities, particularly with other neurodevelopmental disorders, can appear after many years^44, 45^. Therefore, exploring brain development with the help of fNIRS, given the importance of brain development in childhood and adolescence, can be crucial to understand the cognitive and social functioning resulting from the neuroplastic adaptations presented by these children.

In a complementary way, multimodal studies with the combination of different methods are currently being explored in pediatric populations^44, 46–48^. Future investigations could combine complementary methods such as electroencephalography (EEG) and electromyography (EMG), associated with NIRS, during real-world social contexts and not just in controlled laboratory tasks, where children may experience more difficulties. An example would be to investigate patterns of frequency bands derived from EEG or measure the degree of muscle activation through EMG, associating them with hemodynamic responses in the context of daily activities while the child plays and interacts with another. Moreover, the trajectories of retinal pathologies associated with ZKV can be simultaneously accessed with the potential developmental alterations in resting-state functional connectivity in primary and associative visual networks^49^. But it is important to emphasize that the varying degrees of visual impairment of patients with CZV might be difficult specific paradigms requiring processing or response to visual stimuli and assessing resting state functional-connectivity and large scale functional networks is preferable.

Finally, although it is challenging to investigate the motor and cognitive-behavioral responses of children with CZV during everyday activities (e.g., during physical therapy or cognitive rehabilitation) might better inform treatment. Changes in functional connectivity patterns during day-to-day tasks between the frontal and sensory-motor cortices, for instance, can be explored as an additional metric to assess the effects of treatment^50^. Metrics based on fNIRS can be associated with already used clinical metrics such as performance tests, thereby opening possibilities for exploring this tool as a long-term predictor when assessing the effectiveness of treatments aimed at this specific population.

The cognitive domain and functional brain regions of interest should be considered before selecting the task during study protocols with fNIRS. However, other factors should also be considered when designing an fNIRS study for specific populations, such as clinical characteristics of the sample, desensitization, movement artifacts and environment control.

Factors such as age, education level, cognitive development and variations in mood state may influence the choice of tasks to be employed^51, 52^. Thus, presenting non-linguistic stimuli such as images or physical stimuli may be more indicated for the pediatric population^51^. In this perspective, memory tasks can be used to measure cognitive function and learning processes^53–55^. For example, preclinical data from nonhuman primate model of zika virus infection indicate that the visual paired comparison (VPC) task can be administered to nonverbal subjects with immature motor skills and provide important insight into the measurement of memory postnatal ZKV-infected^49^.

The first point to be considered in our clinical sample refers to the cranial asymmetries associated with microcephaly. Although the reduced head circumference may seem to be a minor factor requiring only a cap of specialized size, the cranial deformities and discrepancies deserve further attention when setting up the cap. In this way, individualized caps become an imperative factor, and not only the size, but also the material which must allow adequate elasticity and comfort, must be considered.

In terms of comfort, another aspect to be considered is the high rate of irritability reported by this clinical population. Among neurological disorders, continuous irritability and excessive crying are frequent presentations in the follow-up studies of children with CZV^56^. This characteristic can be very challenging, since once crying starts it usually has a long duration which can last for hours, and which therefore requires planning the sessions with a longer time to anticipate solving complications and the child’s return in a calmer state to resume the exam. Fatigue caused by excessive crying and high irritability should also be considered in this context by rescheduling the exam and/or experiment sometimes being indicated in these situations. Considering strategies such as performing the collection right after feeding or administering patients’ routine medications and even during sleep can be useful when planning sessions.

## Conclusion

Children with microcephaly and congenital zika often have associated disorders such as West syndrome, arthrogryposis and changes in muscle tone^11, 57^. Therefore, proper medication management must be observed in view of these challenges, making it possible to reduce the presentation of muscle spasms and convulsive episodes during the session. In a complementary way, the promotion of cervical control can be performed through mechanical support, static surface or manual assistance. In addition to these factors, task specificity which will be conducted during the session must consider the severity of the impairment in psychomotor development. The profile of cognitive and behavioral impairment must be assessed at baseline in order to guide individualized strategies for each patient during the experiment conduct. Neonatal ZIKV infection associated with microcephaly causes even more cerebral atrophy, severe motor dysfunction and seizure episodes^58^. This condition becomes more serious along with other behavioral disorders such as cognitive and social deficiencies^59^. Therefore, a specialized clinical evaluation at baseline with cognitive and behavioral tests becomes imperative for proper planning.

## Methods

### Participants

Twenty-seven children between 3 and 4 years of age were recruited from an ongoing longitudinal study at our department since the outbreak period in Brazil (2015–2016). The study was conducted in the Outpatient Unit at the Universidade Federal da Paraíba (UFPB) in João Pessoa, Paraíba State, Brazil, where the children with neurologic impairments were followed up by a pediatrician, psychiatric, nurse and physiotherapist. We included children with confirmed Zika virus infection during pregnancy through serological and/or molecular tests^60, 61^. Microcephaly was defined as an occipitofrontal circumference with more than two standard deviations below the population mean (which is less than the third percentile)^62^. The study protocol was approved by the Ethics Research Committee of the UFPB and written informed consent was provided by parents or guardians for both study participation and image publication. The protocol was carried out in accordance with the guidelines and regulations of UFPB.

We applied a battery of cognitive and behavioral tests and measured changes in hemodynamic response in a sample of 25 children diagnosed with Microcephaly caused by congenital Zika virus infection using a multichannel fNIRS system. The children had a varying impairment degree classification ranging from mild to moderate and severe impairment. The instruments were applied according to the recommendations for each specific age group. Motor functional impairment was scored according to the Gross Motor Function Classification System (GMFCS)^63^. We used the motor subscale of the Bayley Scales of Infant and Toddler Development Third Edition (BSID-III)^64^ for children up to 3 years old, and we used the Movement Assessment Battery for Children Second Edition for children older than three. Children up to 42 months of age were assessed for cognition using the BSID-III cognitive and language subtest scores^64, 65^, while we used the Wechsler Preschool and Primary Scale of Intelligence Third Edition for those aged 4–6 years. Behavioral assessments were performed using the Diagnostic and Statistical Manual of Mental Disorders^66^ and the Child Behavior Checklist^24^.

### Desensitization protocols

A systematized familiarization and desensitization protocol was applied to prepare participants for fNIRS data collection and to minimize possible adverse factors during scanning. Such procedures followed the recommendations of previous studies involving pediatric populations which also present atypical development, ranging from Premack principle (reinforcement) to classification measures^67^.

In our experience, we initially conducted the desensitization process individually. The process was guided according to the specific needs of each child and considering the stage of cognitive-behavioral impairment presented. We classified the children’s neuropsychological profile into three levels according to specific scores in the clinical assessment instruments (Table 1).

**Table 1:** Children’s neuropsychological profile classification.

Level	Neuropsychological profile
Absent/mild	GMFCS score I; BSID-III cognitive, language, motor score > 84, or global IQ > 70; normal hearing; normal vision, and no behavioral abnormalities
Moderate	GMFCS score II–III; BSID-III cognitive, language, motor score 70–84, or global IQ 50–70; seizures controlled with anticonvulsants; hearing deficit compensated by aids; visual deficit with useful vision or behavior problems
Severe	GMFCS score IV–V; BSID-III cognitive, language, motor score < 70, or global IQ < 50; seizures not controlled with anticonvulsants; hearing loss not corrected by aids; no useful vision or pervasive developmental disorder

In addition to this classification, we monitored the emotional state throughout the experiment, with the child’s consent being considered during all the activities. It is important to highlight that tasks should not be imposed, or the use of the fNIRS cap, especially in children with atypical development in which aversive stimuli can elicit prolonged negative behaviors, making any attempt at new collection impossible.

Desensitization for children with mild impairments involved creating a narrative, describing the pre-collection preparation and conduction of the experiments to the child, in addition to using reinforcers with the Premack principle^68, 69^ and simulating the fNIRS experiment. The entire process was interactive, with the narrative development mediated by the child’s responses. This narrative involved the stages to which the child would be submitted and with the addition of toys. The dialogue was repeated several times with the participant verbalizing each step, which ensured their understanding of the procedures and their familiarization with the new stimuli. Adaptations of the Premack principle involved providing the child with their favorite toy whenever he or she experienced fatigue and/or discomfort at some stage, even if the child was not yet finished.

Symptoms such as hyperkinesia, distractibility and communication difficulties are barrier factors for children with moderate impairments, and alternatives were required to perform desensitization. Narration containing the details of the collection procedures was maintained so that the child could anticipate the experiences that they would go through. However, the narrative was designed according to social stories in the model idealized by Gray and Garand^70^ including adaptations for the study population such as low voice intonation and visual-motor cues to reduce stimuli aversive to touch. Thus, the characters in the story were created within a playful setting and with the help of toys, composing a dramatization of the procedures. Tactile and proprioceptive stimuli were performed on toys symbolizing the child, and then on themself. In this way, not only the sensorimotor system would progressively adapt to touch but also the perception of visual cues. Thereby, this process would unfold a simple exploration of the object on possibilities of affective-emotional exchanges in which the child is represented in the toy^71, 72^. The stimuli were directed to key points of body stabilization due to hyperkinesia, and body control was facilitated with the help of the companion who positioned the child in a firm and comfortable posture while the other procedures were performed.

Finally, other desensitization elements were added for those with severe cognitive and behavioral impairment in addition to the strategies listed for children with moderate impairment. These elements involved adaptations in the environment with reduced brightness and limited number of examiners in the room. Such strategies were applied due to the clinical presentations comprising hyperreflexia, irritability and intense crying, emotional lability, reduced social interaction and communication. Voice intonation during the creation of social history reporting the procedures was further reduced; this caution was necessary due to the low threshold at which Moro’s reflex was elicited, which was followed by intense crying and aversion to the continuity of procedures. The stimuli adapting process, including the fNIRS acquisition environment, the face of examiners and toys used as reinforcers was conducted with a longer duration and individualized for each child. The same occurred for the social history, with several repetitions (to a greater degree when compared to participants classified as having lower impairment), with its completion only being reached when the child demonstrated comfort and acceptance of the procedures involving touching and wearing the fNIRS cap through non-verbal language (eye contact and facial expressions directed to the stimulus).

### Experimentation

#### fNIRS instrumentation

The main components of an fNIRS system is a set of bundles with light sources and detectors (optodes) and an integrated hardware for light sources control and detectors signal measurements (Fig. 1A).

This system is connected to a computer with appropriate software to control the hardware and record the data. The optodes are arranged in a grid placed on the scalp according to the brain regions of interest, by interleaving the sources and detectors (Fig. 1B). The optodes are strategically placed in a flexible cap or headband (Fig. 1C). The source optodes are laser or led-based emitters of at least two specific wavelengths at the near-infrared spectrum of light (from 750 to 2000 nm) (Fig. 1D). The signals (in volts) measured by the detectors can be converted in hemodynamic states (relative changes in oxy and deoxyhemoglobin concentration over time) by using the modified Beer–Lambert equation^73^.

The fNIRS channel is defined as the region illuminated by the path of light between one source and detector, which are usually spaced from 20 to 30 mm. The distance between these optodes are related to the deepness aimed to be achieved (the shorter the distance, the more superficial the area sampled). The usual distance of 20 to 30 mm was demonstrated to capture signal from the cortical surface. In addition, most fNIRS systems are capable of acquiring so-called short-distance measurements (distance between source and detector from 8 to 12 mm). These measurements are useful to disentangle between systemic artifacts, represented in the signal of superficial non-brain tissue (i.e., skin, skull, temporal muscles, and meninges), and proper cortical signals. In order to improve spatial accuracy, it is recommended the utilisation of a neuronavigation system combining a 3D spatial digitizer and a structural MRI.

#### Experimental paradigms and results interpretability

fNIRS is particularly suitable to investigate brain function during early development and in clinical populations in which other functional mapping data are difficult or impossible to obtain. Functional magnetic resonance imaging (fMRI), though the most established and widely applied functional neuroimaging tool, presents enormous challenges and limitations during acquisition in infants and children with neurodevelopmental disorders. Inherent features of the experimental setting such as the aversively noisy environment and the necessity to minimize head movement, besides other requirements of engagement to perform specific tasks, significantly constrain the possible applications of fMRI for such populations. However, accumulated knowledge on optimizing experimental paradigms and designs as well as the ongoing advances on data analyses strategies can be readily translated from fMRI to fNIRS data as both techniques rely on neurovascular coupling phenomena^74^.

As with fMRI, fNIRS indirectly reflects localized neurons' activity through transient variations on local metabolic and hemodynamic status^74^. The nature and dynamics of these variations, usually modeled by an Hemodynamic Response Function (HRF), constrain the possible experimental paradigms and designs and their interpretation. Classificaly, functional neuroimaging experiments involve performing specific tasks in blocks of approximately 20 to 30 s interleaved with other specific tasks or control conditions performed while a subject's brain is scanned. These experimental designs are maint to map the brain regions specifically associated with a cognitive construct of interest. Additionally, event-related designs are intended to track hemodynamic changes related to specific cognitive or behavioral events, further expanding functional mapping possibilities. Though constituting powerful tools for investigating the neural correlates of cognitive development, task-related paradigms heavily depend on one's collaborative effort and active engagement with oftenly demanding tasks. Such requirements prevent the application of task-related paradigms to infants. Moreover, the stage of cognitive–behavioral impairment of a child with CZV might also prevent the application of task-paradigm, and this strategy might probably be suitable only for older children with no or mild impairment.

A critical development in neuroimaging methodology, resting-state paradigms have emerged as a powerful way to investigate neurodevelopment^31, 75–77^. Instead of mapping localized neural activity associated with a given cognitive construct or behavior, these paradigms are used to access the functional connectivity between different brain regions. Low frequency spontaneous oscillations of hemodynamic activity associated with underlying neural oscillations provide a basis for determining the functional coupling of brain regions^78–80^ without the need of children's active engagement with demanding tasks. Besides allowing application of functional neuroimaging to infants and children with moderate to severe cognitive-behavioral impairment, fNIRS resting-state paradigms might provide crucial insights regarding the neurodevelopmental trajectories of global or functionally specific brain networks^81^. However, resting-state functional findings were shown to depend on arousal status, being disrupted by sleep intrusions^82^. Recent studies have shown that the use of naturalistic stimuli (e.g. movies) enhance the reproducibility of functional connectivity and large scale functional network findings^83^.

The Inscapes movie was specifically developed to optimize neuroimaging data acquisition in children^84^. Inscapes is a 7-min long original animation consisting of abstract shapes with no scene cuts or sudden alterations, minimizing confounding effects of language, affective and social processing of conventional movies. The absence of abrupt changes in light intensity, figures shapes or sound track make Inscapes particularly suitable to be shown to children with CZV. However, the effects of using naturalistic stimuli for children with neurodevelopmental disorder in general and the influence on different stages of impairments have not been systematically assessed yet^85^.

Mostly commonly used to infer functional connectivity and large scale networks, resting-state paradigms can also inform local brain physiology and, indirectly, neural activity. Quantitative features of hemodynamic slow frequency fluctuations such as amplitude (ALFF) and regional homogeneity (ReHo) can be used as proxies of localized functional activity^34^. Furthermore, resting-state fNIRS experiments can inform variations in local brain physiology, since the hemodynamic response effects on concentrations of oxy and deoxyhemoglobin are measured accurately and independently. In fact, HRF's shape is influenced by brain pathology, drugs with modulating effects on neurovascular coupling, brain region and developmental stage^86, 87^. The decomposition of hemodynamic effects in relative local concentrations of both oxy and deoxyhemoglobin puts resting-state fNIRS in a good position to investigate localized neurophysiological variations in neurodevelopmental disorders.

In sum, resting-state paradigms, potentially enhanced by optimized naturalistic stimuli, offer a suitable and feasible brain functional assessment of children with neurodevelopmental disorders, regardless of the level of impairment. Particularly, resting-state paradigms can be used regardless of the level of visual impairment. The current variety and continuous development and flexibility of analytical strategies for fNIRS resting-state data allow investigations on the development and disturbances of local activity, functional connectivity, specific and global brain networks.

#### Setup and acquisition

There are a number of challenges regarding the acquisition of high quality fNIRS signal. The impact of some artifacts may be reduced by using tailored preprocessing methods after acquisition (described in further details later in this article). In this subsection, we describe some operational issues to be addressed during the data acquisition to improve the signal-to-noise-ratio. Head movement and physiological systemic artifacts are the main difficulties to be handled in this population, due to both the early developmental stage and clinical impairments.

Undoubtedly, the desensitization procedures described in the previous section is the main crucial step to improve the signal quality. This step is relevant not only in the case of microcephaly patients but in any fNIRS acquisition involving infants and children. Particularly, anxiety associated with biomedical instrumentation and head manipulation often lead to excessive head motion during the experiment. Moreover, the physiological response associated with the resulting arousal may induce systemic artifacts such as greater variability in cardiac and breath rhythms and systolic/diastolic pressure. Though these problems cannot be fully eliminated, significant mitigation can be achieved by proper desensitization protocols.

In addition, the child must remain seated on her mother's (or caregiver) lap during the whole experiment. First, this is fundamental to guarantee the patient safety, which is always the main priority. Second, the patient may touch the headcap or pull the optodes bundle, compromising the collection or damaging the fNIRS system. Third, the caregiver can naturally constrain the patient's hands avoiding movement and unexpected touches on the stimuli presentation device. Finally, the physical contact with the caregiver is also helpful in reducing anxiety and arousal.

The adult should sit in a comfortable armchair large enough to hold the child. All optodes bundle and system cables should be placed out of the patient's sight (ex: from behind the armchair and over the adult's shoulder) (Fig. 1E).

In order to reduce head movement, the child may hold a large teddy bear or cushion to support the chin, which is also helpful to constrain the field-of-vision to the presentation device. Another point of caution is the headcap positioning. The main challenges are not only the expected small head size due to microcephaly but also the cranial anatomy, which may be irregular and asymmetric. These anatomical abnormalities may result in a low adherence of the cap to the scalp causing instability of the optodes support. Thus, depending on the patient, cap arrangements (e.g.: local reduction using strings or rubber-bands) may be necessary. Finally, the layout design of optodes placement is not straightforward. Due to the frequent excessive hair volume of these patients, prefrontal montages are easier to set up. However, depending on the research question, the prefrontal cortex may not be the main target region to be investigated.

## Data Availability

The materials and data used in this study are stored in a private repository, but can be requested at any time to the corresponding author.
